# *De novo* characterization of the pine aphid *Cinara pinitabulaeformis* Zhang et Zhang transcriptome and analysis of genes relevant to pesticides

**DOI:** 10.1371/journal.pone.0178496

**Published:** 2017-06-01

**Authors:** Songqing Wu, Zhicheng Huang, Carballar-Lejarazú Rebeca, Xiaoli Zhu, Yajie Guo, Qiannan Lin, Xia Hu, Rong Wang, Guanghong Liang, Xiong Guan, Feiping Zhang

**Affiliations:** 1 College of Forestry, Fujian Agriculture and Forestry University, Fuzhou, People’s Republic of China; 2 Fujian-Taiwan Joint Center for Ecological Control of Crop Pests, Fujian Agriculture and Forestry University, Fuzhou, People’s Republic of China; 3 Key Laboratory of Biopesticide and Chemical Biology, Ministry of Education, Fujian Agriculture and Forestry University, Fuzhou, People’s Republic of China; 4 College of Life Science, Fujian Agriculture and Forestry University, Fuzhou, People’s Republic of China; 5 Department of Biology and Biotechnology, University of Pavia, Pavia, Italy; 6 State Key Laboratory of Cellular Stress Biology, Innovation Center for Cell Signaling Network, School of Life Sciences, Xiamen University, Xiamen, Fujian, China; Universita degli Studi della Basilicata, ITALY

## Abstract

The pine aphid *Cinara pinitabulaeformis* Zhang et Zhang is the main pine pest in China, it causes pine needles to produce dense dew (honeydew) which can lead to sooty mold (black filamentous saprophytic ascomycetes). Although common chemical and physical strategies are used to prevent the disease caused by *C*. *pinitabulaeformis* Zhang et Zhang, new strategies based on biological and/or genetic approaches are promising to control and eradicate the disease. However, there is no information about genomics, proteomics or transcriptomics to allow the design of new control strategies for this pine aphid. We used next generation sequencing technology to sequence the transcriptome of *C*. *pinitabulaeformis* Zhang et Zhang and built a transcriptome database. We identified 80,259 unigenes assigned for Gene Ontology (GO) terms and information for a total of 11,609 classified unigenes was obtained in the Clusters of Orthologous Groups (COGs). A total of 10,806 annotated unigenes were analyzed to identify the represented biological pathways, among them 8,845 unigenes matched with 228 KEGG pathways. In addition, our data describe propagative viruses, nutrition-related genes, detoxification related molecules, olfactory related receptors, stressed-related protein, putative insecticide resistance genes and possible insecticide targets. Moreover, this study provides valuable information about putative insecticide resistance related genes and for the design of new genetic/biological based strategies to manage and control *C*. *pinitabulaeformis* Zhang et Zhang populations.

## Introduction

Sooty mold is a devastating disease in pine trees [1] excreted by the sucking aphid *C*. *pinitabulaeformis* Zhang et Zhang, together with direct damage that aphids produce to pine needles results in economical loss in Asia. In China, *C*. *pinitabulaeformis* Zhang et Zhang is geographical distributed in several provinces including Liaoning, Beijing, and Shandong; high population levels of this aphid results in broad damage to forest resources, landscapes and has an impact in the ecological environment of China. Aphids, both nymphs and adults, feed exclusively on plant phloem sap [2] by inserting their mouthparts and then inject saliva that might be phytotoxic [1]. In addition, aphids can transmit several viruses: 275 out of 600 (nearly 50% of insect-borne viruses) which could cause many harmful diseases [3, 4]. Therefore, effective control strategies for *C*. *pinitabulaeformis* Zhang et Zhang are critical steps to protect the pine trees, playing an important role in treatment and prophylaxis of pine diseases.

At present, the primary strategies to control *C*. *pinitabulaeformis* Zhang et Zhang include: physical control (burning of branches and leaves that contain eggs), chemical control (insecticides), biological control (natural enemies, such as *Coccinella septempunctata* [5], *Adalia bipunctata* [6], *Hyperaspis repensis* [7], *Hippodamia variegate* [8] have shown high effectiveness). Among these, biological control has unique advantages: (1) no (or low) resistance to biological agents, (2) no environmental risk and (3) through genetic modification natural enemies can be used to generate strongly pathogenic strains to overcome pine pests. However, biological control can be expensive and it can be a high risk strategy when introducing higher numbers of other insect populations; moreover, natural enemies may compete and produce toxic substances to inhibit other natural enemies [4]. In the past decade, genetic modification tools offer a new insight in pest control through genetic modification (transgenesis). However, there is currently lacking of knowledge regarding *C*. *pinitabulaeformis* Zhang et Zhang, gene function and gene expression in this insect.

We used next generation sequencing technology to sequence the transcriptome of *C*. *pinitabulaeformis* Zhang et Zhang and successfully built a transcript database. In addition, our data describes putative insecticide resistance genes, olfactory related receptors, stressed-related protein, detoxification related molecules, possible insecticide targets and propagative viruses. This study provides basic valuable information that can be used to develop new genetic based strategies and novel molecular tools to control *C*. *pinitabulaeformis* Zhang et Zhang.

## Results and discussion

### GO assignments

**Fig 1:**
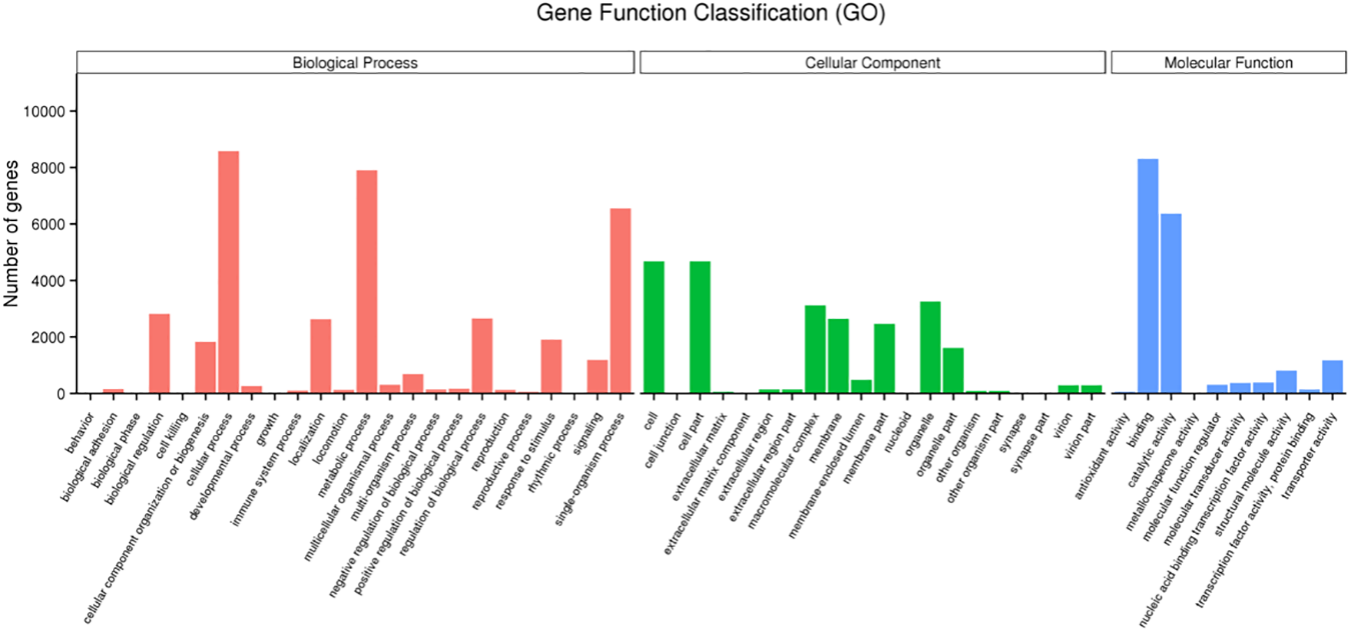
Distribution of second level GO of *Cinara pinitabulaeformis* Zhang et Zhang transcriptome. Distribution of GO categories assigned to the *Cinara pinitabulaeformis* Zhang et Zhang transcriptome. Unigenes were annotated in three categories: cellular components, molecular functions, and biological process. Left y-axis indicates the number of genes in a category.

A total of 80,259 unigenes by BlastX were assigned to GO terms (Fig 1, S1 Table). All unigenes and differential gene expression were classified into three main GO categories (molecular functions, biological processes and cellular components) and 54 subcategories statistically according to the standard GO terms. Biological processes represented most of GO annotations (38,247, 47.65%), followed by cellular components (24,080, 30.00%) and molecular functions (17,932, 22.34%) respectively. Cellular (22.44%), metabolic (20.66%) and single-organism (17.13%) processes were prevalent within biological processes category; with highly emphasis in the following subcategories: biological regulation (7.33%), regulation of biological process (6.93%), localization (6.86%), response to stimulus (4.98%), cellular component organization or biogenesis (4.76%) and signaling (3.11%). Biological processes subcategories include most of the majority cellular processes, such as translation, supersession, transportation and cell formation, transcription, etc. Cellular components category was overrepresented mainly by cell part (19.41%), cell (19.41%) and organelle (13.49%) subcategories. Moreover, we identified genes involved in the synthesis of secondary metabolites, and were grouped into binding (46.36%), catalytic activity (35.46%), transporter activity (6.54%), structural molecule activity (4.47%), nucleic acid binding transcription factor activity (2.17%), molecular transducer activity (2.11%), and molecular function regulator (1.71%). Comparative transcriptome of *Sitobion avenae F* (English aphid) and *Acyrthosiphon pisum* (pea aphid) showed the importance of metabolic pathways [9]. GO annotations describe the general features of *C*. *pinitabulaeformis* Zhang et Zhang gene expression, and suggest that expressed genes encode diverse regulatory, stress and structural proteins.

### COG classification

**Fig 2:**
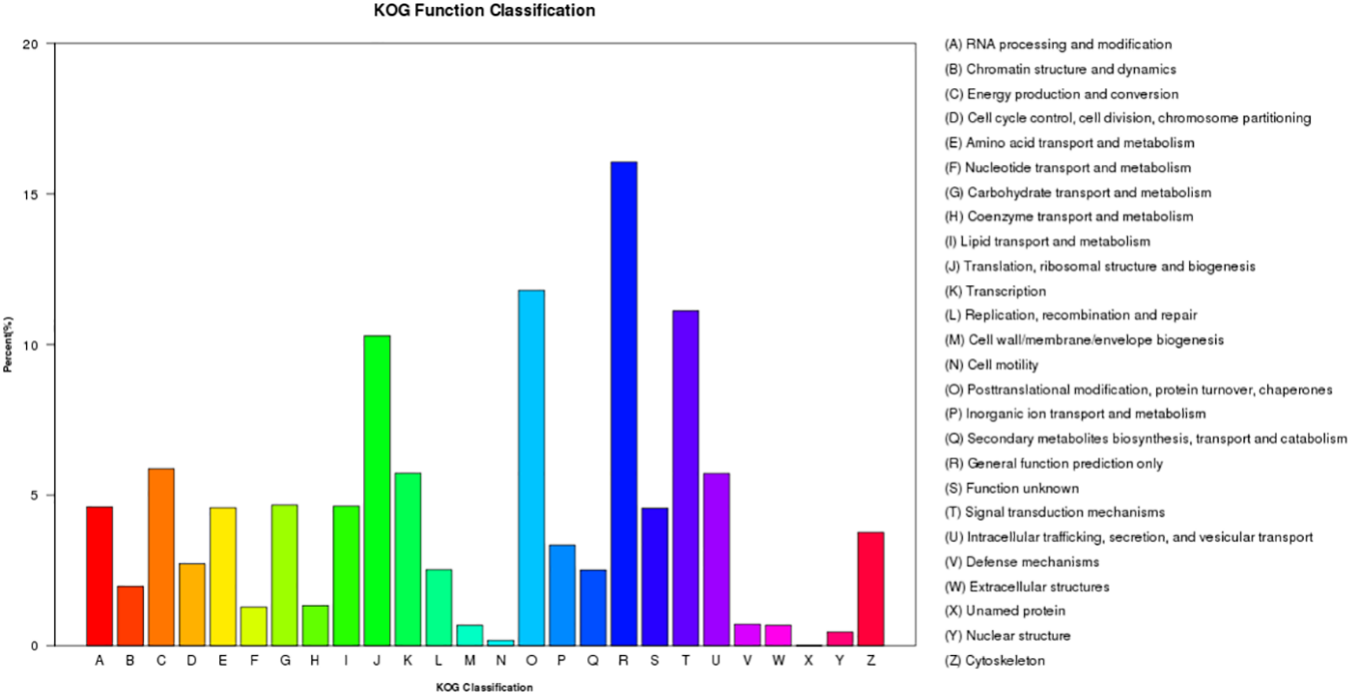
COG function classification of *Cinara pinitabulaeformis* Zhang et Zhang. All putative proteins were analyzed using the COG database. COG classifications were divided into 26 functional categories; 11,609 classified unigenes were assigned to 26 COG classifications.

A total of 11,609 classified unigenes were identified using the COG database (Fig 2) under 26 functional categories. General function prediction (16.06%) was the largest group, followed by posttranslational modification, protein turnover and chaperones (11.80%), signal transduction mechanisms (11.12%), translation, ribosomal structure and biogenesis (10.29%), energy production and conversion (5.87%) and transcription (5.73%). In addition, secondary metabolites biosynthesis, transport and catabolism represented 2.53%, given the importance of secondary metabolic activity for resistance of insects. COG classification can further reveal the biological function and an insight into chemical reactions in molecular processes in *C*. *pinitabulaeformis* Zhang et Zhang.

### KEGG analysis

A total of 10,806 annotated unigenes were analyzed to identify the represented biological pathways in *C*. *pinitabulaeformis* Zhang et Zhang. Briefly, 8,845 unigenes matched with 228 KEGG pathways, summarized in S2 Table. Signal transduction in environmental information processing (1,136 members) was the most highly represented pathway, followed by translation and folding (1022 members), sorting and degradation in genetic information processing (686 members), endocrine system in organismal systems (683), transport and catabolism (642), carbohydrate metabolism (637) and lipid metabolism (439), immune system (408), amino acid metabolism (383), and digestive system (377). KEGG identified pathways can provide new insight in the study of insect biology and contribute to the prediction of higher-level complexity of cellular processes and organism behavior from genomic information.

### Identified viruses

Viruses can be an important tool to control aphid pests, however, it is important to identify the virus transmitted by *C*. *pinitabulaeformis* Zhang et Zhang. For example, Bonning *et al*., used coat protein from luteovirus to deliver insect neurotoxins to create an effective oral toxin, which caused aphid dead after they eat the transgenic plant that contains the protein-toxin fusion [10]. There is a great potential to take advantage of the viruses that are present in *C*. *pinitabulaeformis* Zhang et Zhang. Among the identified virus sequences are reovirus, geminivirus, tymovirus and occlusion-derived virus (S3 Table). Aphid transmission of these viruses may cause plant weakness, yellowish, slow growing and/or death. For example, geminivirus can reprogram plant cell cycle and transcriptional control, by interfering with cell signaling, protein turnover, suppressing immune pathways, and inhibit cell death [11]. As result, transmission of these viruses can have economic impact in crops [12]. Although, plants have many strategies for virus resistance; some viruses rapidly evolve by recombination, mutation, and component capture, allowing these viruses to evade or counter these strategies rapidly [13]. The identified virus can suggest that aphids might be a reservoir for plant virus transmission resulting in plant diseases. Further studies on these viruses may be important to develop new strategies to control other hemipteran pests and may provide information for an efficient strategy to control virus spreading and use of viruses.

### Vitellogenin genes

Four vitellogenin unigenes were identified in of *C*. *pinitabulaeformis* Zhang et Zhang transcriptome. Vitellogenin is the main source of nutrition for the embryo, it has antioxidant activity in honey bee [14, 15]; and it was also reported it has many other biological functions, such as temporal division of labor and foraging specialization, regulation of hormonal dynamics and change in gustatory responsiveness [16, 17]. *Vg* is an important factor for the population proliferation of pest, moreover, aphids have great potential of reproduction; further studies are needed to understand the molecular mechanisms of reproductive biology in *C*. *pinitabulaeformis* Zhang et Zhang, and generate new knowledge on the effect of *Vg* expression patterns in population dissemination.

### Salivary gland related proteins

The saliva in aphids plays important physiological roles in detoxifying toxic substances or in continuous ingestion of the sieve element sap. We identified six laccases and 107 aminopeptidases unigenes in *C*. *pinitabulaeformis* Zhang et Zhang (S4 Table).

Laccases have been detected in the cuticles of many species like *Bombyx mori* and *Schistocerca gregaria* [18] but also in the midgut, Malpighian tubules, fat body and salivary gland [18]. It is suggested that acts as a detoxifying enzyme to overcome the chemical defenses of the host plant (phenolic compounds) [18, 19]. Moreover, laccase knockdown in *Tribolium castaneum* can lead to lethality associated with defects in both pigmentation and cuticle hardening [7].

Aminopeptidase genes are generally expressed in the midgut [20], however, they can also be expressed in the salivary gland [21]. Aminopeptidase-N cleavages amino acids from the amino termini of proteins [22] and have been found to interact with plant-expressed-lectins in *A*. *pisum* [23]. Aminopeptidases expressed in salivary have been commonly detected in aphids, suggesting that they protect from toxic molecules such as plant lectins [6, 24, 25]. The lack of aminopeptidase expression in aphids may avoid aphid to fix on the plant surface, which could contribute in the aphid’s death; aminopeptidase can be a target for genetic manipulation strategies to control aphid pests.

### Stress-related protein

Heat shock proteins (HSPs) are related to stress responses [26], and are divided into five major groups: small HSP (sHsp), HSP60, HSP70, HSP90, and HSP100 [27]. Among these groups, HSP70 family is the largest group of HSPs and can be separated into two sub-groups: HSP70 and heat shock protein cognates (HSC70), based on the expression patterns as response to various stimuli [28]; while HSP70 is inducible and expressed at very low levels under basal conditions; however, its transcription and translation are quickly induced as response to stress [29–31]. In general, HSPs are upregulated by stress conditions [26], and are better known for protecting the cell against thermal stress conditions [32–35]. Moreover, HSPs function as molecular chaperones to promote correct refolding and prevent aggregation of denatured proteins [36]. In *C*. *pinitabulaeformis* Zhang et Zhang we identified sHsp, HSP60, HSP70, HSP90; as well as HSP67B2, HSP68, HSPSTI1, HSP98, HSP110, HSPSSA, HSP cognate 3 (HSC70-3), HSP20, HSP9/12, and HSP-like protein (S5 Table). However, there are not detailed descriptions about these genes in *C*. *pinitabulaeformis* Zhang et Zhang. HSPs can play an important role in temperature tolerance of *C*. *pinitabulaeformis* Zhang et Zhang to survive under stress environmental conditions.

### Olfactory receptors

Volatiles of plant are important signals for recognition by aphids. In insects, such signals bind to odorant binding receptors (OBPs) [37, 38] and then transported to chemoreceptors [39–42], which can activate a cascade of events for sensory neuron activity. OBPs are found in the sensilla lymph, and connect the external environment and odorant receptors (ORs) [37, 38]. ORs play a role in odorant detection and transduction of chemical signals into electric signals [40, 43, 44]. Moreover, chemosensory proteins (CSPs) are also found within the sensilla lymph [45], antennae [46, 47], proboscises [48], legs [49], wings [50], and pheromone glands [46] as well as other tissues. CSPs enhance the solubility of pheromone and delivering them to chemosensory receptors [46, 51].

In general, olfactory protein include ORs, OBPs, CSPs, SNMPs, ionotropic receptors (IRs) and gustatory receptors (GRs), which are related to recognition of host plant and congener species, foraging, sexual behavior, defense, nest mate recognition and caste regulation.

We identified 8 ORs, 14 OBPs, 14 CSPs (8 homologous and 6 putative) and 5 predicted SNMPs in *C*. *pinitabulaeformis* Zhang et Zhang (S6 Table). In contrast, other insects have more olfactory receptors, for example, 43 ORs and 15 OBPs in *Ips typographus* (Ityp) have been identified in *Dendroctonus ponderosae* 49 ORs and 31 OBPs and and 111 ORs in *Tribolium castaneum* (Tcas) were identified [52]. In addition, 6, 11 and 20 CSPs were identified in *I*. *typographus*, *D*. *ponderosae* and *T*. *castaneum* respectively [53]. Our results suggest that olfactory related-genes are conserved in the pine aphid.

In aphids olfactory receptors are needed to recognize pheromones during migration [54]. The understanding of the olfactory systems of *C*. *pinitabulaeformis* Zhang et Zhang can contribute in the development of novel biological control methods [55].

### Calcium channels

Calcium channels play important roles in the transmission of Ca^2+^, which can affect other ion transmission. In *C*. *pinitabulaeformis* Zhang et Zhang a total of 45 calcium channels unigenes were identified and classified into L-, N-, P/Q-, R-, or T-type Ca^2+^ channels based on their electrophysiological and pharmacological properties (S7 Table) [56]. Calcium channels in aphids are targets of the spider insecticide Hv1a. Hv1a is lethal to wide range of insects including aphids, blocking ion passage through the channels or modifying the gating mechanism that controls opening and closing of the ion pore [57].

### Putative insecticide resistance-related genes

#### 1. Cytochrome P450 (P450)

P450 is involved in a number of physiological functions such as adaptability of parasitic plants [58, 59], hormone metabolism [60–62] and resistance to insecticides [63, 64]. Moreover, cytochrome P450 is associated to pyrethroid-, propoxur-, and dichlorvos- insecticides resistance in the mosquito *Culex pipiens* [65]. We identified 69 of P450-related unigenes (S8 Table) and 68 putative P450 genes in *C*. *pinitabulaeformis* Zhang et Zhang. We identified 35 P450-related sequences with a length bigger than 600 bp (24.81% of the global pests in the database); a quarter of the unigenes sequences were long sequences (600bp). Moreover, 17 P450-related sequences with a length larger than 1500 bp (18.05%) (Table 1). P450-related genes identified from *C*. *pinitabulaeformis* Zhang et Zhang were comparable in number with genes from the transcriptome of *Trimeresurus yunnanensis* [66].

**Table 1:** Putative P450 genes identified in *Cinara pinitabulaeformis* Zhang et Zhang.

#Gene ID	Length(bp)	FPKM	E_value	Annotation
c10753_g1	1935	2.13	0	PREDICTED: cytochrome P450 307a1-like [Acyrthosiphon pisum]
c11586_g1	1981	19.38	0	PREDICTED: probable cytochrome P450 6a14 [Acyrthosiphon pisum]
c12584_g1	1592	2.31	0	PREDICTED: probable cytochrome P450 6a13 [Acyrthosiphon pisum]
c14557_g1	2043	7.99	0	PREDICTED: probable cytochrome P450 6a13 [Acyrthosiphon pisum]
c14601_g1	2142	93.64	0	PREDICTED: probable cytochrome P450 6a14 [Acyrthosiphon pisum]
c15263_g1	1570	3.18	5.72E-177	PREDICTED: cytochrome P450 4C1-like [Acyrthosiphon pisum]
c15718_g1	1942	11.33	0	PREDICTED: probable cytochrome P450 6a13 [Acyrthosiphon pisum]
c16009_g1	2159	36.14	0	PREDICTED: cytochrome P450 6k1-like [Acyrthosiphon pisum]
c16118_g1	1825	5.21	0	PREDICTED: cytochrome P450 4C1-like [Acyrthosiphon pisum]
c16811_g1	1882	4.04	0	PREDICTED: cytochrome P450 4C1-like [Acyrthosiphon pisum]
c17063_g1	1899	18.85	0	PREDICTED: probable cytochrome P450 6a13 [Acyrthosiphon pisum]
c22803_g1	1750	2.28	0	PREDICTED: cytochrome P450 6j1-like [Acyrthosiphon pisum]
c27052_g1	2435	719.92	0	PREDICTED: cytochrome P450 4g15 [Acyrthosiphon pisum]
c37250_g1	2519	17.57	0	PREDICTED: cytochrome P450 4C1-like [Acyrthosiphon pisum]
c42684_g1	1507	39.6	0	PREDICTED: probable cytochrome P450 305a1 isoform X1 [Acyrthosiphon pisum]
c8797_g1	1925	40.67	0	PREDICTED: cytochrome P450 18a1 [Acyrthosiphon pisum]
c9000_g1	1978	41.12	0	PREDICTED: probable cytochrome P450 303a1 [Acyrthosiphon pisum]

Based on silkworm P450 gene homology and the phylogenetic analysis of *Drosophila* four large families of P450 enzyme system were identified: CYP2, CYP3, CYP4 and mitochondrial P450 [67].

It has been reported that 17 P450 genes from CYP3 and CYP4 families are associated with pesticide resistance and plant toxin resistance [67, 68]. Based in phylogenic analysis; we found evidence that CYP2, CYP6 and mitochondrial families are present in the transcriptome of *C*. *pinitabulaeformis* Zhang et Zhang, as well as, CYP3/CYP5/CYP6/CYP9 and CYP4/CYP19/CYP26. We also identified CYP6a2, CYP6a13, CYP6a14, CYP6a18, CYP6k1, CYP6CY3 and CYP6B1 belonging to CYP6 family, and CYP4v2 belonging to CYP4 family. Interestingly, the P450 gene families identified in *C*. *pinitabulaeformis* Zhang et Zhang differ from those reported in pea aphid and other insect systems [67, 69].

According to recent studies, P450 possesses resistance-related function to insecticide and plant secretions [70]. There is also evidence that the overlap degree of P450 is related to detoxification and metabolism. However, there is no experimental data to prove the biological role of P450 complex related to insecticide resistance in *C*. *pinitabulaeformis* Zhang et Zhang.

#### 2. Glutathione S-transferase (GST)

GSTs are responsible for detoxification of xenobiotic compounds like plant secondary metabolites and insecticides [71, 72]. High levels of GSTs expression are involved in insecticide resistance in insects [73]. We identified 48 GST unigenes in *C*. *pinitabulaeformis* Zhang et Zhang, 15 of them with a size larger than 1000bp (31.25%) (Table 2). According their cellular location, GSTs are divided into three major categories: microsomal, cytosolic and mitochondrial [74]. Moreover, in insects cytosolic matrix GSTs are further divided into at least six classes (delta, epsilon, omega, sigma, theta, and zeta) which is based on homology of the N-terminus sequence, immunoreactivity, substrate specificity, and sensitivity to different inhibitors [75–77]. Epsilon and delta classes are unique in insects [78]. We identified delta, omega, sigma, theta, zeta types of GST in *C*. *pinitabulaeformis* Zhang et Zhang transcriptome, nevertheless we could not identify any genes from the epsilon class. However, GST delta class is only expressed in wingless aphid like pea aphid, *A*. *pisum* [79]. Sigma, delta and theta GST classes have been identified in other insects such as *Nasonia vitripennis*; one delta unigene in *T*. *yunnanensis*; and epsilon, sigma, omega, and delta in *T*. *castaneum* have been identified [80, 81]. The GST genes identified in the transcriptome of *C*. *pinitabulaeformis* Zhang et Zhang can contribute to further understanding of insecticide resistance mechanisms in aphid pests.

**Table 2:** Putative identified GST genes in *Cinara pinitabulaeformis* Zhang et Zhang.

#Gene ID	Length(bp)	FPKM	E_value	Annotation
c36827_g1	1373	78.55	3.72506E-127	glutathione S-transferase 1-1-like [Acyrthosiphon pisum]
c17698_g1	1153	108.62	9.74E-119	uncharacterized protein [Acyrthosiphon pisum]
c12960_g1	1834	35.13	6.20E-130	glutathione S-transferase omega-1-like [Acyrthosiphon pisum]
c15950_g1	1694	5.4	0	PREDICTED: prostaglandin E synthase 2-like [Acyrthosiphon pisum]
c9303_g1	1445	9.77	5.52E-110	glutathione S-transferase sigma 2 [Aphis gossypii]
c579_g1	1048	9.42	5.55E-47	hypothetical protein YQE_12493, partial [Dendroctonus ponderosae]]
c10389_g2	2491	91.6	1.05E-55	hypothetical protein CAPTEDRAFT_142825, partial [Capitella teleta]
c3297_g1	2747	62.57	0	PREDICTED: failed axon connections [Acyrthosiphon pisum]
c8152_g1	1343	1.98	0	PREDICTED: elongation factor 1-gamma [Fopius arisanus]
c15915_g1	1187	4.04	1.63E-106	glutathione S-transferase, theta class-like [Acyrthosiphon pisum]
c14487_g1	1174	22.37	6.71E-127	PREDICTED: glutathione S-transferase theta-1-like [Acyrthosiphon pisum]
c27263_g1	1089	900.51	5.25E-128	glutathione S-transferase delta 1 [Aphis gossypii]
c2324_g1	1845	38.86	0	elongation factor 1-gamma [Acyrthosiphon pisum]
c12316_g1	2437	2.08	0	glutathione S-transferase, C-terminal domain containing [Acyrthosiphon pisum]
c11123_g1	1328	40.68	0	PREDICTED: chloride intracellular channel exc-4 isoform X1[Acyrthosiphon pisum]

### Other insecticide resistance-related genes and insecticide receptors

**Fig 3:**
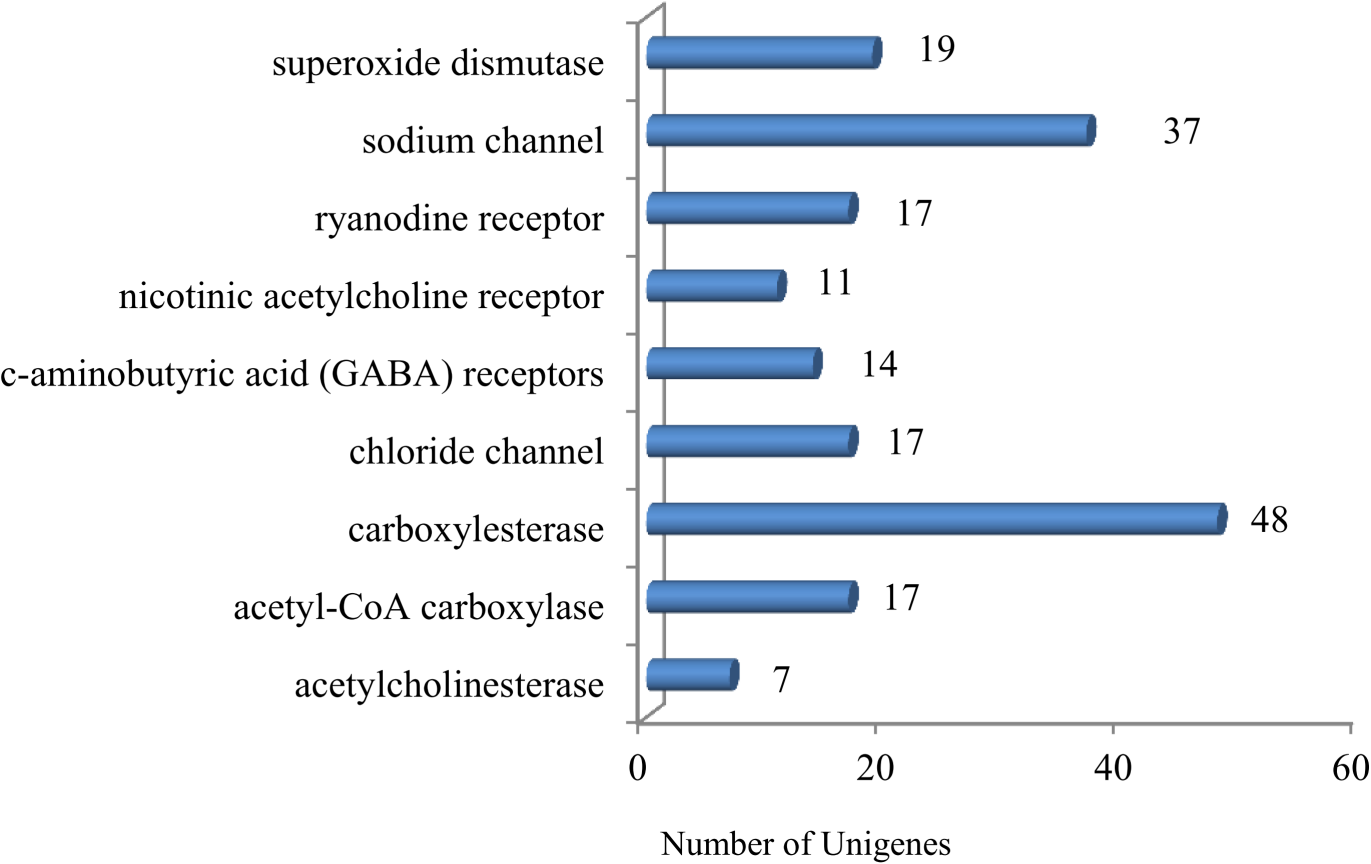
Number of unigenes related to insecticide receptors and resistance-related genes. x-axis indicates the number of unigenes, y-axis indicates the specific unigenes related to pesticide receptors and resistance-related genes. One hundred and eighty-seven insecticide receptors and resistance-related unigenes were identified in *C*. *pinitabulaeformis* Zhang et Zhang transcriptome.

We also identified 187 unigenes (S9 Table) with potential insecticide resistance functions, pesticide receptors and detoxification, such as: carboxylesterase, sodium channel, superoxide dismutase, acetyl-CoA carboxylase, chloride channel, ryanodine receptor, c-aminobutyric acid (GABA) receptors, nicotinic acetylcholine receptor, acetylcholinesterase (Fig 3**)**. These genes can provide basic information to contribute in the knowledge of insecticide mechanism, which are not described in aphids.

### RNA interference-related genes

RNA interference (RNAi) is a mechanism of homology dependent gene silencing present in plants and animals [82]. RNAi is high specificity, efficiency, stable, transmissible and hereditary; this characteristics make it a valuable tool to address various questions in insect toxicology, as well as may become an effective strategy for management of insect pests [83].

**Fig 4:**
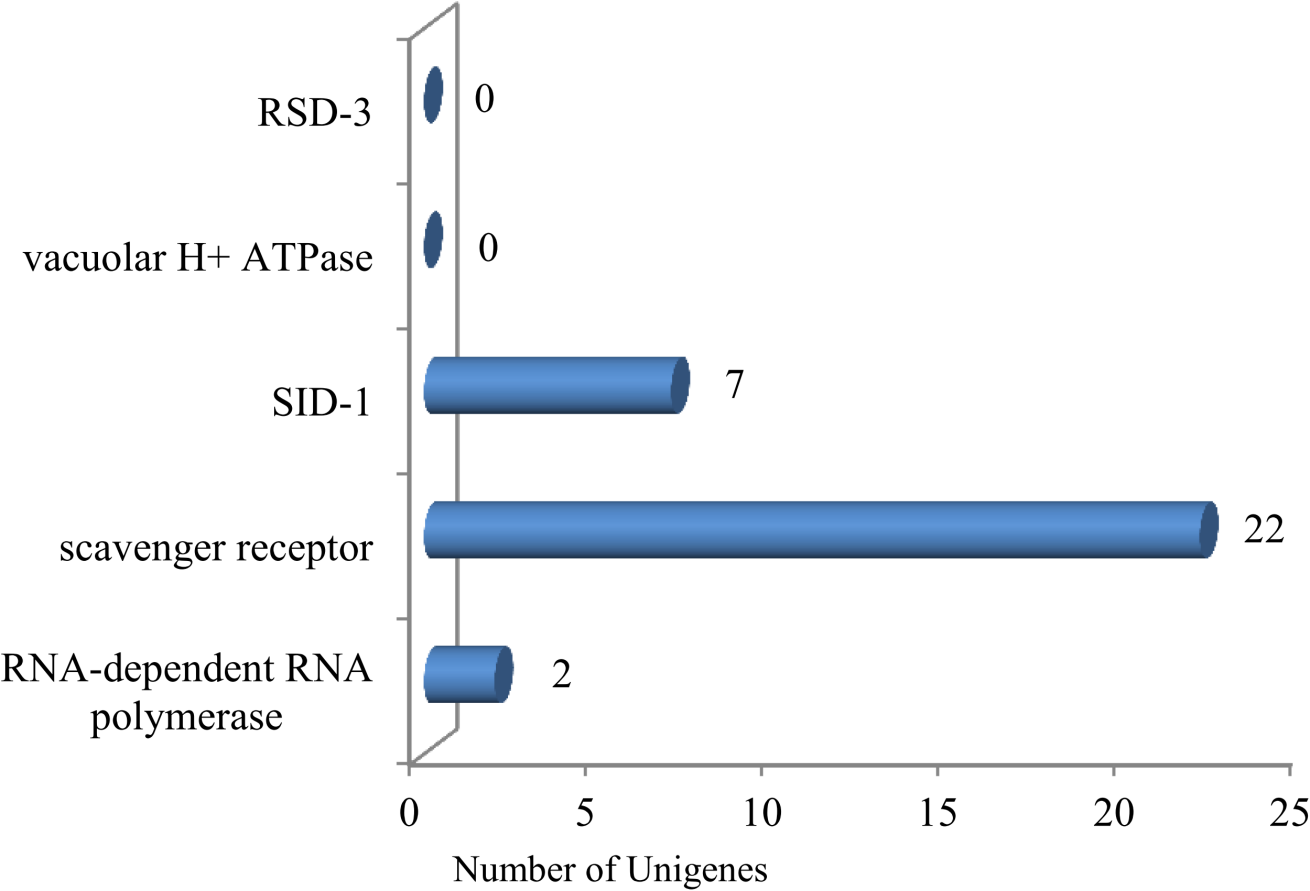
Number of unigenes related to RNAi. x-axis indicates the corresponding number of unigenes and y-axis indicates the specific unigenes related to RNAi. Thirty-one unigenes coding for RNAi identified in the database.

Thirty-one unigenes related to RNAi pathways were identified in *C*. *pinitabulaeformis* Zhang et Zhang (Fig 4): two RNA-dependent RNA polymerases, twenty-two scavenger receptors (SRs), and seven Systemic RNA Interference Defective1 (SID-1) unigenes. These components previously have been reported in insects as part of RNAi uptake mechanisms [84–86]. However interestingly, we did not identify any vacuolar H^+^ ATPase and RSD-3 unigenes, although they are involved in RNAi pathways. Among the identified genes, 20 unigenes were larger than 700bp (64.52%) and 17 were more than 1kb (54.84%). In addition, one SID-1-related unigene and one scavenger receptors were both represented by longer sequences in the transcriptome analysis, at c15255_g2 (4397bp) and c16045_g1 (4065bp), respectively.

Scavenger receptors (SRs) can recognize polyanionic ligands which are extensive and in *Drosophila* [87], they play key parts in mediating phagocytosis of pathogens and immune responses (suppression of scavenger receptors transcription by parasitoid factors). SRs bind to proteins, polysaccharides, polyribonucleotides and lipids directly [88, 89]; recently it has been shown that SRs are involved in microRNA delivery [90]. Among 22 unigenes related to scavenger receptors, thirteen of them had lengths above 1kb, while the rest ranged from 300bp to 1kb.

SID-1 is crucial for systemic RNAi pathways in *Caenorhabditis elegans*, which is a multi-span transmembrane protein; it delivers dsRNAs to cells [87] and it was identified as a required gene for systemic, but not cell-autonomous, RNAi [91]. In the cotton aphid, *Aphis gossypii* a *sid-1* orthologue has also been identified, although this gene has not been identified in *D*. *melanogaster* [92].

Further studies of scavenger receptors, SID-1 and RNA-dependent RNA polymerase genes can increase the understanding in the biology of cell protection against endogenous/exogenous nucleic acids of pathogens. Moreover, understanding the mechanisms of RNAi pathways will contribute to develop new molecular approaches to control plant pathogens and insects through suppression of key genes/proteins of infecting organisms.

## Conclusion

*Cinara pinitabulaeformis* Zhang et Zhang is known as the main pest of pine trees and produce dense dew which lead to the sooty mold which makes pine trees infect with disease. In this work, we sequenced and characterized the transcriptome of *C*. *pinitabulaeformis* Zhang et Zhang by Illumina sequencing. We identified a large group of genes related to propagative viruses, nutrition-related genes, detoxification related molecules, olfactory related receptors, stressed-related protein, putative insecticide resistance genes and possible insecticide targets. This study provides valuable information that may play an important role in the developing new control strategies to manage *C*. *pinitabulaeformis* Zhang et Zhang populations.

## Materials and methods

### Ethics statement

There are no specific permits for insect collection in the selected locations. The sampling locations are not privately-owned or natural protected areas. Insects used for the experiments are not considered endangered or protected species, and its collection is legal in China.

### Insects

Wingless adult aphids of *C*. *pinitabulaeformis* Zhang et Zhang were collected from *Pinus massoniana* (*P*. *massoniana*) branches in the town of JinJiang, ZiMao mountain and FuJian province (N 26.15046°; E 119.59261°). Ten aphids (five females and five males) were stored at -80°C until RNA extraction.

### Illumina sequencing and cDNA library

Total RNA was extracted from whole aphid adults using TrizolH (Invitrogen, USA). Residual genomic DNA was removed by incubation with DNase I, Amp Grade (Invitrogen, USA) and RNA integrity was evaluated by agarose gel electrophoresis. RNA was quantified on a NanoDrop ND-1000 spectrophotometer (Thermo Scientific, USA). For RNA sample preparations, a total amount of 1.5 μg RNA per sample was used as starting material. Generation of sequencing libraries was done using NEBNext® Ultra™ RNA Library Prep Kit for Illumina® (NEB, USA), following the manufacturer’s recommendations. Briefly, purification of mRNA from total RNA was done using poly-T oligo-attached magnetic beads. Fragmentation was performed by using divalent cations under high temperature in NEBNext First Strand Synthesis Reaction Buffer (5x). First strand cDNA was synthesized using random hexamer primers and M-MuLV Reverse Transcriptase (RNase H-). Second cDNA strand was generated using DNA Polymerase I and RNase H. The residual overhangs were converted into blunt ends using exonuclease/polymerase. NEBNext Adaptor contained a hairpin loop structure was ligated for hybridization after adenylation of the 3’ ends of DNA fragments. In order to select cDNA fragments of 150~200 bp in length, the purification of cDNA fragments were done using the AMPure XP system (Beckman Coulter, Beverly, USA). Three μl of USER Enzyme (NEB, USA) was used in conjunction with cDNA which was size-selected, adaptor-ligated at 37°C for 15 min followed by 5 min at 95°C before PCR reaction. PCR reaction was performed using Phusion High-Fidelity DNA polymerase, Universal PCR primers and Index (X) Primer. PCR products were purified (AMPure XP system) and library quality was assessed using the Agilent Bioanalyzer 2100 system.

### Bioinformatic analysis

The cDNA library was sequenced by High-throughput sequencing platform which was allowed to produce many high-quality reads based on Sequencing by Synthesis (SBS) technology. Raw data were from cleaned low-quality reads and joint sequences. The identified transcript sequences in each fragment collection, was done using the De Bruijn method of graphing the sequencing read information [93]. E-value of the BLAST parameter was set at 10^−5^, and E-value of the HMMER parameter was set at 10^−10^. All unigenes were compared with Swiss-Prot [94], GO [95], NR [96], COG [95], KEGG [97], KOG [98] and databases using BLAST [99] software. COG and KEGG databases were used to predict metabolic pathways. The function of the identified transcript and assignment of Gene Ontology (GO) terms were determined using the GO database. The output of amino acid sequence of unigenes was analyzed using the HMMER [100] software and searched in the Pfam [101] database to gain annotated information for the unigenes.

## Supporting information

S1 Table Gene Ontology of *Cinara pinitabulaeformis* Zhang et Zhang unigenes. (XLS)

S2 Table KEGG summary of *Cinara pinitabulaeformis* Zhang et Zhang transcriptome. (XLS)

S3 Table Identified viruses in *Cinara pinitabulaeformis* Zhang et Zhang transcriptome. (XLS)

S4 Table Secreted protein genes identified in *Cinara pinitabulaeformis* Zhang et Zhang transcriptome. (XLS)

S5 Table Heat shock protein genes identified in *Cinara pinitabulaeformis* Zhang et Zhang transcriptome. (XLSX)

S6 Table Olfactory receptor related genes identified in *Cinara pinitabulaeformis* Zhang et Zhang transcriptome. (XLS)

S7 Table Calcium channel identified in *Cinara pinitabulaeformis* Zhang et Zhang transcriptome. (XLS)

S8 Table Putative P450 genes identified in *Cinara pinitabulaeformis* Zhang et Zhang transcriptome. (XLS)

S9 Table Insecticide receptors and resistance-related identified genes in *Cinara pinitabulaeformis* Zhang et Zhang transcriptome. (XLSX)
